# A hybrid STL–LightGBM framework with probabilistic forecasting for Influenza A incidence in the post-pandemic Saudi Arabia

**DOI:** 10.3389/fpubh.2026.1803353

**Published:** 2026-04-10

**Authors:** Reham M. Alahmadi, Maaweya Awadalla, Bashayer Saeed, Huda M. Alshanbari, Alshaikh A. Shokeralla, Ali Atif Yassin, Bandar Alosaimi, Fathelrhman El Guma

**Affiliations:** 1 Department of Botany and Microbiology, College of Science, King Saud University, Riyadh, Saudi Arabia; 2 Research Center, King Fahad Medical City, Riyadh Second Health Cluster, Riyadh, Saudi Arabia; 3 Department of Mathematical Sciences, College of Science, Princess Nourah bint Abdulrahman University, Riyadh, Saudi Arabia; 4 Department of Mathematics, Faculty of Science, Al-Baha University, Al-Aqiq, Saudi Arabia; 5 Department of Statistics, Faculty of Science, University of Tabuk, Tabuk, Saudi Arabia

**Keywords:** Influenza A, STL decomposition, LightGBM, probabilistic forecasting, non-stationary time series, structural regime shifts, epidemiological surveillance

## Abstract

Influenza A outbreaks in Saudi Arabia exhibit different seasonal patterns, influenced by significant changes, including a near-total halt during the COVID-19 pandemic (2020-2021) and a substantial rebound, as evidenced by national surveillance data, until the end of 2023. Traditional time-series models rely on stationarity and stable seasonal patterns; however, these assumptions are significantly undermined by regime shifts. This study introduces a forecasting method that uses light gradient boost machine (LightGBM) regression, along with Seasonal-Trend decomposition using LOESS (STL), to better track influenza in changing contexts. The proposed method adapts to the evolving epidemiological dynamics shaped by policy and behavioral changes by decomposing the incidence series into long-term trends, stable annual seasonal components, and irregular residual fluctuations prior to nonlinear learning. Exploratory analysis supports strong winter seasonality, linear correlations with meteorological variables, and major structural disruptions linked to pandemic-related interventions. shows how standard SARIMAX and seasonal baseline models cannot be used across all epidemiological regimes. The hybrid model, when evaluated during the test window, shows strong out-of-sample performance, substantially outperforming the benchmark models (*R*^2^ = 0.831, MAE = 89.0). In-sample fitting throughout the study period indicates a high degree of representational capacity (*R*^2^ = 0.987). The framework is further extended to probabilistic forecasting via quantile regression, resulting in accurately calibrated 95% prediction intervals. The uncertainty in the predictions increases appropriately during periods of epidemiological disruption, highlighting the importance of uncertainty-aware prediction under structural change. The proposed STL-LightGBM architecture is a resilient and comprehensible instrument for monitoring influenza in post-pandemic contexts, facilitating early warning systems and expeditious public health decision-making in Saudi Arabia and analogous regions.

## 1 Introduction

The COVID-19 pandemic has profoundly altered the global epidemiology of seasonal influenza through an unprecedented combination of public health and social measures (PHSMs), including mobility restrictions, mask mandates, school closures, and changes in healthcare-seeking behavior. Indicators point to a fact that these interventions did not merely temporarily halt the spread of influenza but possibly disrupted the natural pattern of its seasonal spread, with a consequent delay and totally irregular post-pandemic activity of the virus across different geographical settings. A recent multi-regional study of eight regions across the world reported significant departures from pre-pandemic seasons of influenza spread after the removal of interventions aimed at controlling COVID-19, with increased temporal irregularity and heterogeneous resurgence rather than any signs of returns to normal and previous seasonality (1). Concurrent with these findings, supportive surveillance results reported from the United States showed historically low levels of influenza activity during the seasons of 2020–2021, with essentially asynchronous and virus-specific resurgence after the removal of pandemic control interventions (2).

At the national level in Saudi Arabia, there remains convergent evidence of a significant decrease in influenza activity during its pandemic years. A multicenter time series study demonstrated a large decrease in laboratory-diagnosed influenza cases during 2020–2021, coinciding with an escalation in severe public interventions and behavioral modifications (3). Furthermore, evidence of significant effects of COVID-19 interventions on influenza dynamics in Saudi Arabia arises from a national-level epidemiologic research that showed mitigation of COVID-19, in particular, altered its trend of time-dependent variations in influenza in Saudi Arabia, reaching its lower level during severe interventions and rising after alleviating interventions (4). Convergently, knowledge, attitudes, and practices of Saudis concerning seasonal influenza vaccination have been demonstrated in behavioral research, providing clear evidence of COVID-19 pandemic effects in changing knowledge, attitudes, and practices and making SIV vaccination behaviors, underscoring the importance of behavioral considerations of influenza in post-pandemic periods in Saudi Arabia (5). Collectively, these studies establish a robust epidemiological context in which influenza transmission in Saudi Arabia has been substantially disrupted by the COVID-19 pandemic, even though publicly available monthly surveillance series remain limited.

Despite extensive progress in seasonal influenza forecasting, many conventional modeling approaches remain rooted in assumptions that have been increasingly challenged in the post-pandemic era. Classical models, such as ARIMA, SARIMA, SARIMAX, and the Holt-Winters method, tend to require quasi-stationarity of the system as well as seasonal cycles remaining strong in nature. Although this assumption might hold in the pre-2020 world, the performance of the models is shown through the contradicting evidence that, under pandemic suppression, resurgence, and out-of-season epidemic peak conditions, the models fail (6). In addition, “analyses of historical influenza-like illness counts suggest that, even well-tuned models of this type tend to suffer in reliability in the face of abrupt policy changes and population-level intervention behaviors” (6).

To address nonlinear transmission dynamics and complex temporal dependencies, recent post-pandemic forecasting studies emphasize the significance of comparative assessment of statistical and machine learning methods under diverse epidemiological circumstances. Baker et al. revealed that LSTM and ARIMA exhibit consistent and adaptable performance across several regional COVID-19 datasets, highlighting the significance of model selection, training duration, and contextual data attributes in epidemic forecasting (7). machine learning and deep learning techniques—such as long short-term memory networks (LSTM) and gradient-boosting frameworks—have been increasingly applied to influenza forecasting, including within the Saudi context (8, 9). These approaches have demonstrated improved point forecast accuracy relative to purely linear baselines; however, they are predominantly deterministic and often provide limited quantification of predictive uncertainty. This limitation is particularly consequential in the post-COVID-19 landscape, where influenza dynamics are shaped by stochastic behavioral responses, fluctuating PHSMs, and residual interactions with other respiratory viruses (10). As a result, strong point accuracy alone may obscure substantial uncertainty, potentially leading to overconfident projections when forecasts are used to inform public health preparedness and resource allocation.

This concern is further substantiated by the results of recent epidemiological investigations showing significant disruptions of traditional respiratory virus seasons during and after the SARS-CoV-2 pandemic. Indeed, heterogeneous re-emergence of respiratory viruses among different age groups, temporal shifts of specific viral seasons, and desynchronized re-emergence of influenza and other respiratory pathogens after lifting NPIs are found (2, 5). Stochastic and fractional-order models of influenza epidemics are particularly useful tools for analyzing the role of random fluctuations, memory effects, and parameter uncertainty in shaping the course of such epidemics and demonstrating their leading role–including during periods of rapid epidemiological dynamics (11–13). Simultaneously, the aforementioned list of observations points to an important methodological limitation of traditional deterministic frameworks of influenza forecasting, both statistical and machine learning-based. Indeed, such tools are found inappropriate for incorporating effectively the aforementioned characteristics of emerging post-pandemic seasons of infectious diseases.

Influenza incidence time series are inherently non-stationary, exhibiting strong seasonality, abrupt regime changes, and transient anomalies that challenge purely linear modeling assumptions. Decomposition-based approaches have therefore gained prominence as a means of separating complex signals into interpretable components prior to forecasting. Wavelet transform techniques, in particular, offer time—frequency localization that facilitates multiscale decomposition of trend, seasonal, and irregular components, thereby enhancing robustness under changing dynamics (14). Building on this rationale, recent influenza-specific studies demonstrate that integrating wavelet-based decomposition with probabilistic learning frameworks, such as Gaussian Process Regression (GPR), can outperform classical baselines while simultaneously providing uncertainty-aware forecasts of direct relevance to public health decision-making (15). More broadly, hybrid modeling strategies—combining linear statistical structures with nonlinear learners—have emerged as a pragmatic response to the limitations of single-model approaches. Hybrid architectures such as ARIMA—GRU and related formulations have been shown to improve predictive performance by leveraging complementary strengths in capturing short-term structure and nonlinear dependencies (16, 17). Decomposition-driven hybrids have also consistently demonstrated advantages over standalone ARIMA models in non-stationary outbreak settings, reinforcing the value of signal separation prior to prediction when the data-generating process evolves over time (18).

Beyond model architecture alone, recent reviews emphasize that contemporary epidemic forecasting increasingly integrates non-traditional data sources and interpretability mechanisms. Comprehensive surveys highlight the growing role of Internet-derived signals, digital surveillance streams, and auxiliary covariates in enhancing outbreak detection and short-term forecasting, although these elements are often incorporated in isolation rather than within cohesive modeling pipelines (19). Insights from large-scale COVID-19 forecasting initiatives further indicate that sustained predictive performance is strongly associated with model designs that explicitly account for regime shifts, behavioral adaptation, and cumulative stochastic error, rather than relying solely on black-box accuracy improvements (20). Within this dynamic context, boosting schemes driven by decomposition have demonstrated a quite good performance. Recent research targeted at influenza demonstrates that the incorporation of signal decomposition with learners based on LightGBM and residual correction methods can significantly enhance the forecasting accuracy of rapidly changing conditions in the transmission of infection (21). Cross-country evidence in COVID-19 forecasting backs that smoothing and seasonal—trend decomposition methods, including STL, facilitate enhanced forecast stability from more varied modeling families, even where model choice remains the key determinant of accuracy (22). For benchmarking purposes, the classical baselines represented by ARIMA remain irreplaceable, as a series of comparative analyses identified for this review repeatedly reveals deficiencies inherent in using pure linear assumptions about highly irregular healthcare demand and epidemic time series (23).

In parallel, the integration of external information sources and interpretable feature selection has gained increasing attention. Deep recurrent models augmented with Internet-derived indicators, such data from Google Trends and the Baidu Index better carry out early detection and perform short-term forecasting when combined with systematic feature selection and optimization strategies (24, 25). Although not influenza-specific, spatially resolved dengue forecasting across Brazil further illustrates the value of integrating lagged climatic drivers, spatial dependencies, and SHAP-based interpretability to identify dominant predictors and temporal delays, providing a transferable methodological rationale for explainable covariate modeling in infectious disease forecasting (26). Similarly, hybrid multi-component architectures developed in environmental domains, such as air quality prediction, support the broader principle that combining short- and long-term pattern extractors with attention mechanisms and post-hoc interpretability can enhance generalization under extreme and heterogeneous conditions (27).

### 1.1 Study objectives and proposed approach

Motivated by these considerations, this study endeavors to advance influenza forecasting in a post-pandemic context by means of a probabilistic hybrid framework inspired by time-series decomposition. The concrete objectives pursued in this work are: (i) characterizing the degree of nonstationarity and structural disruption present in the monthly Influenza A surveillance series analyzed in the present study (2017–2025), as a premise for regime-aware and decomposition-based modeling; (ii) benchmarking the predictive performance and robustness of classical statistical baselines comprising SARIMAX and seasonal naïve models against modern machine learning approaches under post-pandemic conditions; (iii) developing a hybrid forecasting framework integrating seasonal–trend decomposition with LightGBM to capture nonlinear dynamics while retaining interpretability; and (iv) extending the proposed framework to probabilistic forecasting with the explicit quantification of predictive uncertainty. Combining decomposition-aware modeling, boosting-based learning, and uncertainty quantification into a single pipeline, the proposed approach seeks to yield a robust and interpretable tool for influenza surveillance and public health decision-making in settings characterized by regime changes and increased uncertainty.

## 2 Materials and methods

### 2.1 Data sources and preprocessing

Laboratory-confirmed monthly cases of influenza A were accessed from the FluNet database of the Global Influenza Surveillance and Response System (GISRS), supported by the World Health Organization (WHO) (28). This system acts as a standardized global influenza epidemiological surveillance system providing nationally aggregated influenza case counts from the influenza laboratories accredited across the globe. It has been widely employed for the surveillance of influenza and also for the *time*−*series* models of respiratory viral infection.

The series spans from January 2017 to June 2025, resulting in 102 monthly measurements. The length of the series captures different phases, ranging from typical influenza activity to the pandemic period, as well as the post-pandemic period. The resulting series displays unusual statistical properties, including extended zero-event lengths, particularly within 2020–2021, where 12 months of zero cases resulted from non-pharmaceutical interventions implemented during the pandemic, as well as unusual maximum value behavior, particularly within October 2023, where the series displays 1,570 cases, reflecting an unprecedented post-pandemic rebound.

In addition to influenza incidence data, monthly climatic variables were retrieved from the Worldwide Energy Resources Prediction Database (POWER) provided by the National Aeronautics and Space Administration (NASA) (29). The variables include average air temperatures (°C), relative humidity (%) and total precipitation (mm). The NASA POWER system offers information from satellite measurements with ground-based meteorological measurements and provides globally consistent climate data at a spatial resolution of up to 0.5 degrees, making it suitable for large-scale regional analyzes.

Climatic data were extracted for the main geographical extent of Saudi Arabia and temporally aligned with the Influenza A time series at a monthly resolution. Monthly averages for all climatic variables were used to reduce short-term daily variability and to focus on seasonal patterns and long-term trends relevant to the dynamics of influenza transmission.

Data preprocessing involved converting the original year and month fields into a continuous monthly time index using the first day of each month (01-MM-YYYY) to ensure chronological consistency. The data set was verified to be complete and without missing observations. No outliers or extreme values were removed or adjusted, in order to preserve genuine epidemiological signals, including the zero-incidence months during the COVID-19 period, which are critical for representing abrupt structural changes in the underlying epidemiological system. Given the aggregated monthly nature and completeness of the data, no additional statistical preprocessing procedures were required prior to analysis.

### 2.2 Exploratory data analysis and time-series decomposition

#### 2.2.1 Baseline models

Autoregressive integrated moving average (ARIMA) models are classical statistical tools widely used for linear time-series forecasting. These models are designed to accommodate temporal dependence through the inclusion of autoregressive, moving-average, as well as differencing terms. Unlike exponential smoothing techniques, which explicitly decompose trend and seasonal patterns, ARIMA-based approaches rely on linear stochastic representations to describe the underlying data-generating process (30–32).

Despite their popularity, ARIMA models present notable practical limitations. In particular, selecting appropriate model orders is often subjective and challenging, especially when dealing with complex or noisy data (33). Furthermore, standard ARIMA formulations perform poorly when applied to seasonal time series, as they lack explicit mechanisms for capturing periodic behavior (33). To address this shortcoming, the ARIMA framework was extended to the Seasonal ARIMA (SARIMA) model, which incorporates both seasonal and non-seasonal components, thereby allowing improved representation of recurring patterns in univariate time series (34, 35).

The SARIMA model is characterized by non-seasonal orders (*p, d, q*) and seasonal orders (*P, D, Q*)_*s*_, where *s* denotes the length of the seasonal cycle. Let *Y*_*t*_ represent the observed value at time *t*. The general SARIMA formulation can be expressed as


$$\begin{array}{l} \alpha_{p}\left(L\right)\alpha_{P}\left(L^{s}\right){\left(1-L\right)}^{d}{\left(1-L^{s}\right)}^{D}Y_{t}=\beta_{q}\left(L\right)\beta_{Q}\left(L^{s}\right)u_{t}, \end{array}$$ (1)

where *L* denotes the lag operator, *u*_*t*_ is a zero-mean error term, and α(·) and β(·) represent autoregressive polynomials and moving-averages, respectively.

The non-seasonal autoregressive and moving-average components are defined as


$$\begin{array}{l} \text{AR: }\alpha_{p}\left(L\right)=1-\alpha_{1}L-\alpha_{2}L^{2}-\cdots -\alpha_{p}L^{p}, \end{array}$$ (2)

$$\begin{array}{l} \text{MA: }\beta_{q}\left(L\right)=1-\beta_{1}L-\beta_{2}L^{2}-\cdots -\beta_{q}L^{q}, \end{array}$$ (3)

while the seasonal autoregressive and moving-average components are given by


$$\begin{array}{l} \text{SAR: }\alpha_{P}\left(L^{s}\right)=1-\alpha_{1}L^{s}-\alpha_{2}L^{2s}-\cdots -\alpha_{P}L^{Ps}, \end{array}$$ (4)

$$\begin{array}{l} \text{SMA: }\beta_{Q}\left(L^{s}\right)=1-\beta_{1}L^{s}-\beta_{2}L^{2s}-\cdots -\beta_{Q}L^{Qs}. \end{array}$$ (5)

### 2.3 Autoregressive models with exogenous variables

To overcome the limitations of purely univariate modeling, autoregressive models with exogenous variables were also considered. The ARIMAX model extends the ARIMA framework by incorporating external explanatory variables alongside the autoregressive and moving-average terms, effectively combining time-series modeling with multiple linear regression (36). This formulation allows additional covariates to influence the evolution of the target series.

The ARIMAX model can be expressed as


$$\begin{array}{l} Y_{t}=\mu +\overset{m}{\underset{i=1}{\sum }}\theta_{i}L^{\ell_{i}}X_{t}+u_{t}, \end{array}$$ (6)

where *X*_*t*_ denotes the exogenous input series, θ_*i*_ are regression coefficients, ℓ_*i*_ are lag orders, and *u*_*t*_ is the error term.

### 2.4 Seasonal ARIMAX (SARIMAX) model

The SARIMAX model further generalizes SARIMA by integrating exogenous variables while retaining a seasonal structure. By allowing external covariates to enter the model, SARIMAX aims to improve predictive performance, reduce residual autocorrelation, and provide a more informative multivariate baseline (37). The SARIMAX formulation can be written as


$$\begin{array}{l} \alpha_{p}\left(L\right)\alpha_{P}\left(L^{s}\right){\left(1-L\right)}^{d}{\left(1-L^{s}\right)}^{D}Y_{t}=\underset{k}{\sum }{\text{λ}}_{k}Z_{k,t}+\beta_{q}\left(L\right)\beta_{Q}\left(L^{s}\right)u_{t}, \end{array}$$ (7)

where *Z*_*k, t*_ denotes the *k*-th exogenous variable and λ_*k*_ is its associated coefficient.

### 2.5 Autocorrelation analysis

Autocorrelation diagnostics were conducted using the autocorrelation function (ACF) and the partial autocorrelation function (PACF), which are fundamental tools for identifying linear dependence in time-series data. The ACF quantifies the overall correlation across time lags, while the PACF isolates the direct contribution of individual lags after controlling for intermediate dependencies. Together, these diagnostics support the identification of the model, reveal seasonal periodicities, and guide the specification of autoregressive and moving-average components (38, 39).

### 2.6 Stationarity assessment

Stationarity was assessed using the Augmented Dickey–Fuller (ADF) test, a standard unit root test to evaluate whether a time series exhibits constant statistical properties over time (24, 35). The test examines the null hypothesis of a unit root against the alternative hypothesis of stationarity, with inference based on the test statistic and corresponding p-values. The ADF regression can be expressed as


$$\begin{array}{l} \text{Δ}Y_{t}=c+\kappa t+\rho Y_{t-1}+\overset{r}{\underset{j=1}{\sum }}\psi_{j}\text{Δ}Y_{t-j}+u_{t}. \end{array}$$ (8)

Although differencing can be applied to induce stationarity, it is recognized that unit root tests may be sensitive to structural breaks and regime changes. Consequently, the ADF results were interpreted in conjunction with visual inspection and epidemiological context rather than being based on in isolation (6, 10).

### 2.7 Proposed hybrid STL-LightGBM framework

To accommodate pronounced non-stationarity, structural regime shifts, and nonlinear dynamics in post-pandemic influenza transmission, a hybrid forecasting framework integrating Seasonal-Trend decomposition using LOESS (STL) with Light Gradient Boosting Machine (LightGBM) regression is proposed. Decomposition-based hybrid models have been widely shown to improve robustness, stability, and interpretability in non-stationary time-series forecasting by isolating systematic components from irregular fluctuations prior to nonlinear learning (14, 21, 22).

Let ${\left\{Y_{t}\right\}}_{t=1}^{T}$ denote the monthly incidence series of Influenza A. Using STL, the series is decomposed additively as


$$\begin{array}{l} Y_{t}=T_{t}+S_{t}+R_{t}, \end{array}$$ (9)

where *T*_*t*_ represents the smooth long-term trend component, *S*_*t*_ denotes the seasonal component that captures stable annual periodicity (*s* = 12), and *R*_*t*_ is the remaining term that reflects short-term irregular fluctuations and structural disturbances. The STL framework, originally introduced by Cleveland et al., enables flexible seasonalcik estimation of seasonal and trend components and has been extensively applied to non-stationary epidemiological and environmental time series (14).

Robust STL estimation is adopted to mitigate the influence of extreme observations associated with pandemic-related disruptions and post-pandemic rebounds, which are known to bias classical seasonal decomposition methods (22).

Rather than applying machine learning directly to the raw incidence series, the decomposed components are used to construct a structured feature space that stabilizes learning under non-stationary conditions. Specifically, the input feature vector at time *t* is defined as


$$\begin{array}{l} {\text{X}}_{t}=[T_{t-1:t-p},S_{t-1:t-p},R_{t-1:t-p},{\text{Z}}_{t}], \end{array}$$ (10)

where *p* denotes the maximum lag order and **Z**_*t*_ includes climatic covariates, Fourier seasonal terms, and a post-pandemic regime indicator. Similar decomposition-guided feature constructions have been shown to improve learning stability and predictability under regime changes (18, 21).

The conditional expectation of Influenza A incidence is modeled using LightGBM as


$$\begin{array}{l} {Ŷ}_{t}=f_{\text{LGBM}}\left({\text{X}}_{t}\right), \end{array}$$ (11)

where *f*_LGBM_(·) denotes a gradient boosting decision tree ensemble. LightGBM represents the predictive function as an additive expansion


$$\begin{array}{l} f_{\text{LGBM}}\left(\text{X}\right)=\overset{M}{\underset{m=1}{\sum }}\eta g_{m}\left(\text{X}\right), \end{array}$$ (12)

where *g*_*m*_(·) are regression trees, *M* is the number of boosting iterations, and η is the learning rate controlling shrinkage. This formulation enables efficient learning of nonlinear interactions and regime-dependent effects while maintaining strong generalization under limited sample sizes (40, 41).

By integrating STL-based signal decomposition with boosting-based nonlinear regression, the proposed framework explicitly separates stable seasonal structure and long-term evolution from irregular epidemic shocks prior to learning. Recent studies demonstrate that such decomposition-guided hybrid architectures consistently outperform standalone statistical and machine learning models in highly non-stationary outbreak settings (22, 42, 43).

### 2.8 Probabilistic forecasting extension

Although point forecasts provide a single best estimate of future influenza incidence A, they are insufficient for public health decision-making under post-pandemic conditions characterized by structural breaks, increased volatility, and regime-dependent uncertainty. In such settings, probabilistic forecasting is increasingly recommended to quantify forecast uncertainty and support risk-aware preparedness planning (19, 20).

To extend the proposed STL—LightGBM framework beyond deterministic prediction, a probabilistic forecasting layer based on quantile regression is adopted. Instead of modeling only the conditional mean *E*(*Y*_*t*_∣**X**_*t*_), the objective is to estimate conditional quantiles of the predictive distribution,


$$\begin{array}{l} Q_{\tau }\left(Y_{t}|{\text{X}}_{t}\right),\;\tau \in \left(0,1\right), \end{array}$$ (13)

where *Q*_τ_ denotes the τ-th conditional quantile of Influenza A incidence given the predictor vector **X**_*t*_.

Within the LightGBM framework, quantile regression is implemented by minimizing the asymmetric pinball loss function,


$$\ell_{\tau }(y,\hat{y})=\{\begin{array}{ll} \tau (y-\hat{y}), & y\geq \hat{y},\\ (1-\tau )(\hat{y}-y), & y<\hat{y}, \end{array}$$ (14)

which produces consistent estimates of conditional quantiles without imposing parametric assumptions on the underlying error distribution (8). Separate models are trained for each target quantile τ, allowing the predictive distribution to adapt flexibly to heteroskedasticity and asymmetric uncertainty.

In this study, the prediction intervals are constructed using the estimated lower and upper quantiles at τ = 0.025 and τ = 0.975, respectively, forming a prediction interval of 95%,


$$\begin{array}{l} {\text{PI}}_{0.95}\left(t\right)=\left[Q_{0.025}\left(Y_{t}|{\text{X}}_{t}\right),Q_{0.975}\left(Y_{t}|{\text{X}}_{t}\right)\right]. \end{array}$$ (15)

This nonparametric approach avoids distributional assumptions such as Gaussian residuals, which are often violated in epidemic time series that exhibit heavy tails, excess zeros, and abrupt post-intervention rebounds.

Recent studies emphasize that probabilistic forecasting is particularly critical in post-COVID epidemiological contexts, where historical patterns no longer provide reliable variance estimates and uncertainty itself becomes regime-dependent (20, 22). Hybrid models based on decomposition combined with uncertainty-sensitive forecasting have been shown to produce better calibrated prediction intervals than classical ARIMA-based approaches, especially under structural disruption (18).

By integrating quantile-based probabilistic forecasting into the STL-LightGBM framework, the proposed approach provides not only accurate point predictions but also realistic uncertainty bounds that widen appropriately during periods of epidemiological instability. This extension enhances the model's practical value for early warning surveillance, hospital preparedness, and resource allocation in the post-pandemic influenza era.

### 2.9 Evaluation metrics

Model performance was evaluated using a set of complementary accuracy measures commonly adopted in infectious disease forecasting studies characterized by non-stationarity, structural breaks, and heterogeneous outbreak dynamics. Consistent with recent hybrid modeling applications in epidemiology, a multi-metric evaluation strategy was adopted to avoid reliance on a single precision criterion and to capture distinct aspects of predictive behavior (44).

The mean absolute error was considered a primary scale-dependent metric for quantifying the average absolute deviation between observed and predicted incidence. The MAE has been widely used in epidemic forecasting due to its robustness against extreme values, and it offers direct interpretability for case counts, making it particularly suitable for public health decision-making in resource-limited and data-volatile settings (45).

To penalize explicitly large forecast errors associated with the epidemic surges, RMSE was also reported. The RMSE gives a greater weight to extreme deviations and has been informative in the evaluation of the hybrid and wavelet-based models applied to climate-sensitive infectious diseases where abrupt peaks and heavy-tailed behavior are common (46).

The coefficient of determination (*R*^2^) was included as a relative performance measure to assess the explanatory adequacy relative to a naive benchmark. Although *R*^2^ can exhibit negative values under severe regime changes, this behavior has been reported and interpreted as informative in post-intervention epidemiological forecasting, not as a deficiency of the model itself (47).

In combination, these metrics yield a balanced evaluation of average predictive accuracy, sensitivity to extreme outbreak events, and total explanatory power. Such balancing enables robust comparisons among models of forecasting across the evolving epidemiological regimes.

## 3 Results

This section presents the empirical findings of the study, beginning with an exploratory characterization of Influenza A time series and its key structural properties, followed by a comparative evaluation of the baseline statistical and hybrid machine-learning models. Emphasis is placed on assessing model behavior under pronounced non-stationarity, structural regime shifts, and post-pandemic volatility. Results are reported in terms of both point forecast accuracy and probabilistic uncertainty, with particular attention to performance across distinct epidemiological phases identified through expanding-window validation. Collectively, the analyzes above give an integrated evaluation of the suitability, reliability, and clarity of the model based on the development dynamics of the flu. The evaluation of forecasts for January to June 2025 was performed using a recursive one-step-ahead expanding-window methodology, in which predictions were produced sequentially based solely on the information available up to each forecast origin.

### 3.1 Exploratory data analysis and time-series decomposition

Table 1 summarizes the descriptive statistics of the influenza incidence series and associated climatic variables. The Influenza A series exhibits substantial variability and pronounced right skewness, as reflected by the large discrepancy between the mean (121.13) and the median (51.50), along with an extreme maximum of 1,570 cases. These features signal the emergence of epidemic shocks and structural disturbances, especially during the post-pandemic recovery period. The presence of several months with zero incidences further substantiates regime shifts linked to COVID-19 actions.

**Table 1:** Descriptive statistics of the study variables.

Variable	Mean	Std	Min	Q1	Median	Q3	Max
Influenza A	121.13	203.10	0.00	11.25	51.50	152.25	1,570.00
Temperature (°C)	27.09	8.59	12.15	19.44	26.93	34.19	41.58
Relative Humidity (%)	46.24	19.73	10.71	30.46	46.67	65.25	79.54
Precipitation (mm)	31.26	19.62	0.57	14.70	31.23	47.60	68.89

Conversely, climate variables exhibit low variability and stable distributions, indicating that the magnitude of the observed pandemic fluctuations cannot be adequately explained by weather-related factors alone. The descriptive statistics indicate that the influenza incidence series is significantly non-stationary and has heavy-tailed characteristics, necessitating the application of decomposition-based and nonlinear modeling techniques.

**Figure 1:**
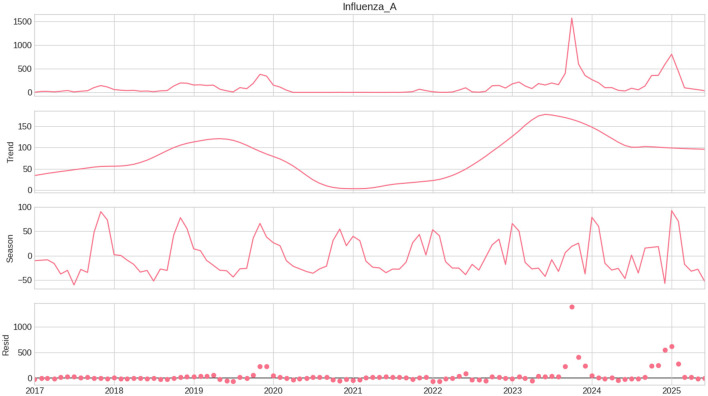
Robust STL decomposition of monthly Influenza A cases. The panels show the observed series, long-term trend, seasonal component, and residual component. Large positive residuals during 2023-2024 indicate abrupt deviations not captured by regular seasonality or trend.

The monthly incidence of Influenza A in Saudi Arabia from January 2017 to June 2025 demonstrates clear annual seasonality, with recurrent winter peaks and substantial temporal variation (Figure 1). Influenza activity was mostly controlled during 2020–2021 due to pandemic-related non-pharmaceutical measures, followed by a significant resurgence in late 2023, peaking at 1,570 recorded cases in October 2023, and thereafter declining throughout 2024–2025.

**Figure 2:**
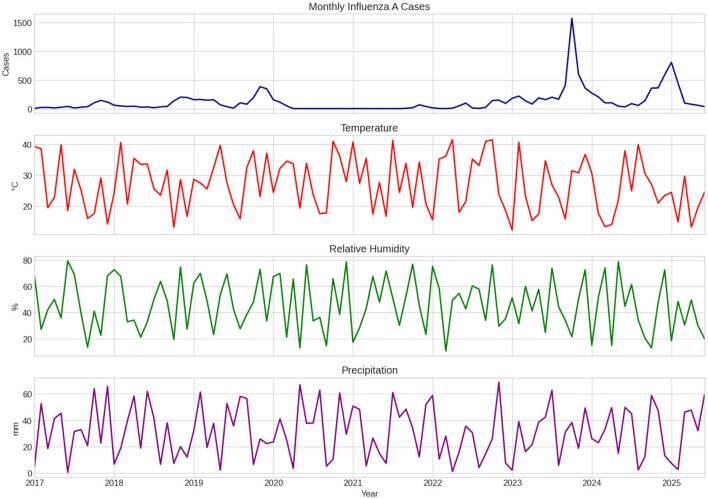
Monthly Influenza A incidence in Saudi Arabia from January 2017 to June 2025 together with selected climatic variables. The series shows pronounced winter seasonality, near-complete suppression during the COVID-19 period (2020-2021), and a sharp post- pandemic rebound within the analyzed dataset in late 2023.

Visual analysis reveals partial inverse comovement between temperature and relative humidity; yet, no substantial concurrent linear correlations are detected. To elucidate long-term patterns, seasonal dynamics, and irregular fluctuations, robust STL decomposition was employed inside each training window to prevent information leaking from future observations (Figure 2).

The decomposition reveals a notable decline in the trend component from 2020 to 2021, followed by a rapid recovery in 2022 and a subsequent steady stabilization. The seasonal component shows persistent winter peaks, affirming a robust seasonal pattern, whereas the residual component displays multiple significant positive spikes in 2023–2024, indicating abrupt variations not accounted for by typical seasonal patterns or long-term trends. These patterns provide empirical evidence of nonlinear dynamics and structural shocks in post-pandemic influenza transmission.

### 3.2 Baseline SARIMAX modeling

As a classical statistical model for evaluation, a Seasonal Autoregressive Integrated Moving Average model with exogenous variables (SARIMAX) was used for linear evaluation purposes. In particular, time series models from the SARIMAX family have demonstrated considerable utility in epidemiological studies. However, it should be noted that their effectiveness depends on several presumptions, including linearity, weak stationarity, and a relatively consistent data-generating process.

Before specifying the models themselves, the stochastic behavior of the Influenza series was investigated using the Augmented Dickey-Fuller (ADF) test. However, even though the ADF test rejected the null hypothesis that the original series contains a unit root (the calculated ADF statistic is (ADF statistic = −4.745, *p* < 0.001), so this should be done with caution. The series exhibits pronounced structural breaks driven by external interventions, including near-complete suppression during 2020–2021 and a sharp post-pandemic rebound within the analyzed dataset. Such regime-dependent dynamics are well known to distort conventional unit-root tests and may yield misleading inferences regarding stationarity.

As such, differencing was undertaken not only for the sole purpose of meeting the formal statistical criteria but also to somewhat dampen the effects of structural breaks. It considered both non-seasonal differencing at frequency (*d* = 1) and seasonal differencing at lag 12 (*D* = 1). Their combined differenced series met the ADF stationarity criterion (ADF statistic = −4.466, *p* < 0.001), which justified a differenced SARIMA-based model as a baseline approach.

Model orders were selected through automated stepwise minimization of the Akaike Information Criterion (AIC). Among the candidate specifications, the SARIMAX(0, 1, 2) × (0, 1, 1)_12_ model yielded the lowest AIC (AIC = 1035.33) and was retained. All estimated moving-average parameters were statistically significant (*p* < 0.05), indicating adequate representation of short-term and seasonal linear dependencies.

**Figure 3:**
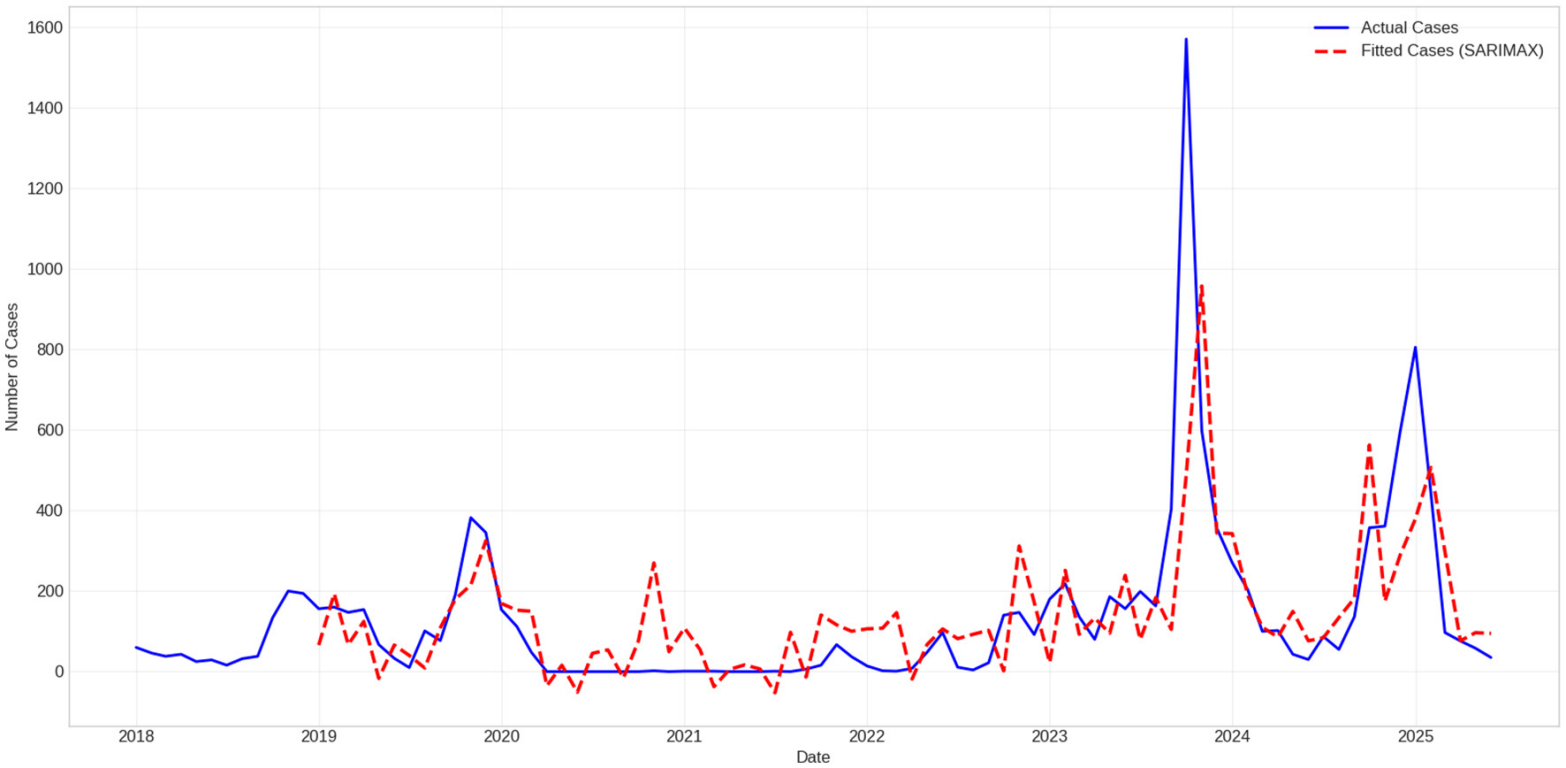
Baseline SARIMAX fit of monthly Influenza A cases. The model captures overall seasonality but underestimates extreme post-pandemic fluctuations and fails to adapt to abrupt structural changes.

Figure 3 compares the baseline SARIMAX fit with the observed series. Although this model accurately meets the major annual seasonality and medium-term fluctuations, it significantly reduces the degree of extreme fluctuation. In addition, it does not adapt to sudden changes in regimes. As seen in the data set used in this model, the substantial increase in late 2023 is severely underestimated because linear-Gaussian models are poorly suited to heavy-tailed data.

Quantitatively, the SARIMAX model explains less than half of the observed variance in the data, with the performance in the sample remaining limited (*R*^2^ = 0.369, MAE = 87.38), indicating a reduced descriptive adequacy under highly volatile epidemic dynamics. Out-of-sample performance during the stabilized post-pandemic test period remains modest (MAE = 194.1, RMSE = 219.2, *R*^2^ = 0.402), despite the inclusion of exogenous climatic variables.

Because only modest gains can be achieved with exogenous input, it supports the view that climatic factors alone cannot account for extreme epidemic fluctuations under post-intervention rebound conditions within a linear SARIMAX framework. Rather, unresolved irregular components, nonlinear effects, and evolving transmission regimes predominate the incidence dynamics of Influenza.

As such, these baseline outcomes from the SARIMAX model indicate that classical linear seasonal modeling is limited in handling non-stationarity, disruptions, and heavy tails, warranting a decomposition-based hybrid modeling approach. For additional benchmarking, a Seasonal Naïve model with a 12-month lag was considered as a simple seasonal reference baseline. This approach assumes that influenza incidence at time *t* is equal to the observed value for the same month in the previous year (*Y*_*t*−12_), thus representing a persistence-based seasonal forecasting strategy commonly used in epidemiological surveillance.

Despite its simplicity, the Seasonal Naïve model provides an important lower-bound reference for evaluating forecasting skill under strong annual periodicity. However, its performance deteriorated under post-pandemic regime changes, producing substantially higher prediction errors than those of the proposed hybrid framework (MAE = 137.2, RMSE = 239.5, *R*^2^ = 0.285 during January–June 2025).

The results demonstrate that merely replicating previous seasonal patterns fails to account for sudden structural disruptions and nonlinear epidemic resurgences evident in post-pandemic influenza dynamics.

### 3.3 Temporal dependence and climatic relationships

Moreover, these results receive support from autocorrelation diagnostics. Indeed, the autocorrelation function shows statistically significant peaks in lags 12, 24, and 36, confirming strong annual seasonal behavior in our time series, while the partial autocorrelation function shows strong dependence in lag 1. Pearson correlation analysis reveals weak contemporaneous linear associations between Influenza A incidence and climatic variables, with absolute correlation coefficients below 0.15.

The strongest lagged association is observed for precipitation with a three-month delay (*r* = 0.143), whereas temperature and relative humidity exhibit only marginal delay effects. These weak linear associations further motivate modeling frameworks capable of capturing nonlinear interactions and irregular dynamics beyond classical regression-based approaches.

### 3.4 Model performance

Model performance was evaluated at three complementary levels: (i) out-of-sample evaluation in the final test window (January–June 2025), (ii) in-sample reconstruction throughout the study period (2017–2025), and (iii) probabilistic calibration using prediction interval coverage. Detailed numerical results are provided in Tables 2 and 3.

**Table 2:** Model comparison on the test period (January–June 2025).

Model	MAE	RMSE	*R* ^2^
Hybrid STL+LightGBM	89.0	116.6	0.831
SARIMAX	194.1	219.2	0.402
Seasonal Naïve (lag 12)	137.2	239.5	0.285

**Table 3:** Full-period in-sample performance comparison.

Model	MAE	RMSE	*R* ^2^
Hybrid STL + LightGBM	6.7	21.3	0.987
SARIMAX	87.38	168.79	0.369
Seasonal Naïve (lag 12)	134.18	259.82	−0.327

The LightGBM model's settings were adjusted via grid search for each training period to improve its ability to adapt to changing disease patterns. Model performance was evaluated using an expanding-window framework with recursive one-step-ahead forecasting.

**Figure 4:**
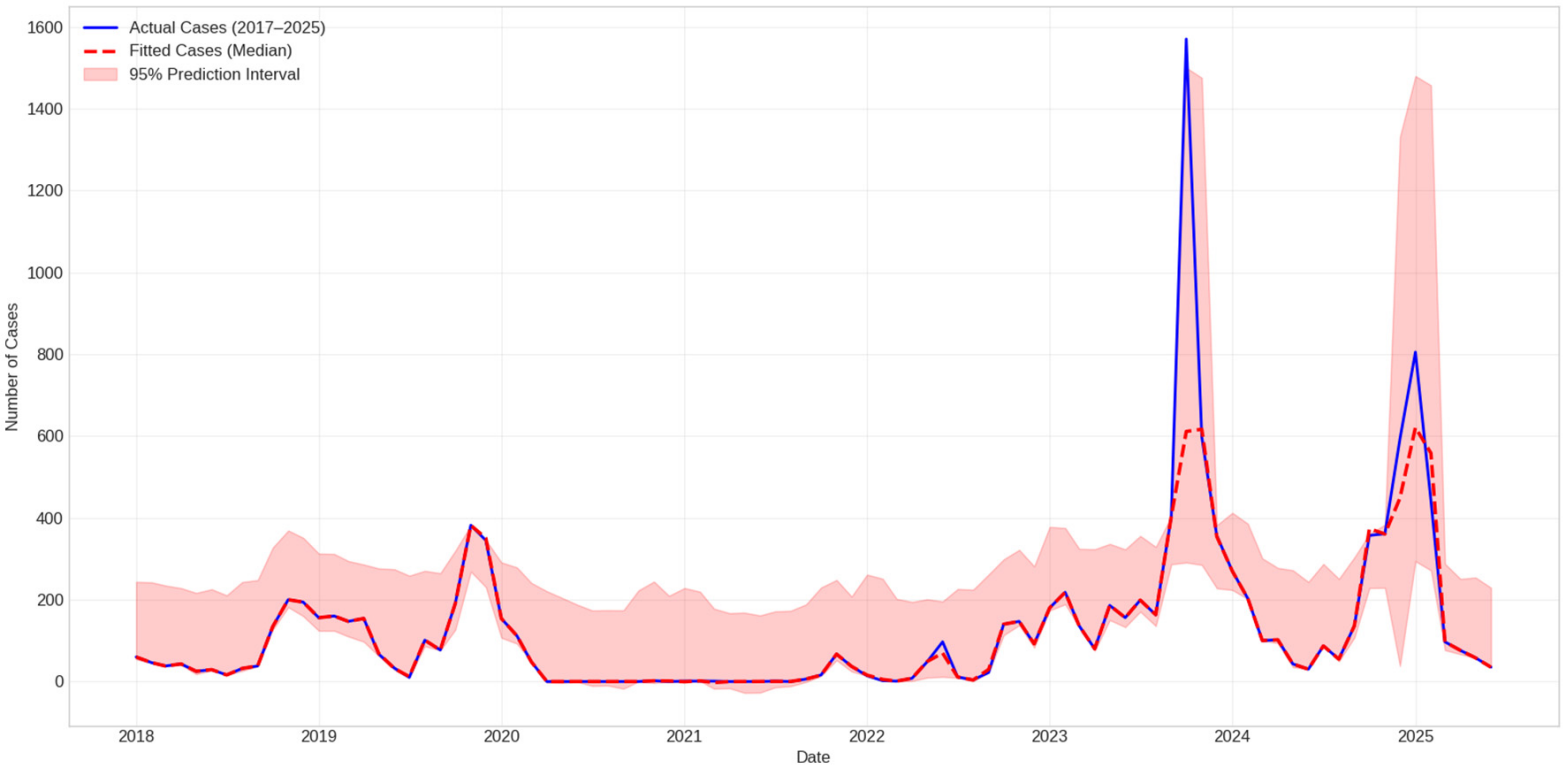
Post-Winter decline in early 2025.

The concluding evaluation period, which signifies a settled post-pandemic phase, was examined in detail. During this period, the hybrid STL-LightGBM model yielded an MAE of 89.0, RMSE of 116.6, and *R*^2^ = 0.831, capturing the observed post-winter decline in early 2025 (Figure 4).

For comparison, the SARIMAX baseline produced higher forecast errors during the same evaluation period (MAE = 194.1, RMSE = 219.2, *R*^2^ = 0.402), while the seasonal naive benchmark demonstrated limited predictive performance under structural changes (MAE = 137.2, RMSE = 239.5, *R*^2^ = 0.285).

A short-horizon multi-step evaluation was also conducted for the final window to examine performance across lead times. Forecast errors increased progressively with longer horizons, whereas one-step forecasts remained comparatively stable.

### 3.5 Probabilistic forecasting and uncertainty quantification

In addition to forecast accuracy, the prediction uncertainty has been evaluated using the 95% prediction interval. Moderate uncertainty is observed during the stable seasonal phases, but it increases noticeably during the epidemiological disruptions, especially during the “COVID-19 suppression” and “COVID-19 post-pandemic rebound” periods. The “extreme peak observed in the late 2023” lies near the upper tail of the predictive distribution, highlighting the advantages of probabilistic prediction of structural regime shifts. The empirical coverage of the 95% prediction intervals in the evaluation folds was approximately 0.96.

### 3.6 Feature importance

Feature importance analysis based on gains shows that the most influential predictors are the autoregressive Influenza A terms at lags 1 and 12, the post-COVID indicator, followed by the Fourier terms capturing the annual seasonality of both contaminants, as well as the absolute humidity. Climatic variables contribute primarily as secondary modifiers. Gain-based importance is used here as a global indicator; future work may extend interpretation through SHAP-based local explanations to further enhance model interpretability.

## 4 Conclusion

The study created and assessed a hybrid forecasting model for monthly Influenza A incidence in Saudi Arabia. It integrates Light Gradient Boosting Machine (LightGBM) regression with Seasonal-Trend decomposition utilizing LOESS (STL). The methodology was developed to tackle non-stationary dynamics and structural regime shifts evident in post-pandemic surveillance data by extracting long-term trends, seasonal patterns, and irregular residual components before nonlinear modeling.

Exploratory analysis indicated strong annual seasonality, near-complete suppression of influenza activity during the COVID-19 intervention period (2020–2021), a post-pandemic resurgence in late 2023, and a subsequent decline during 2024–2025. Weak contemporaneous associations with climatic variables, together with the presence of structural changes, suggest limitations for conventional linear time-series models under such evolving conditions.

The expanding-window evaluation highlighted the difficulty of forecasting across epidemiological regimes. Early folds showed reduced predictive performance when extrapolated to post-pandemic conditions, whereas the hybrid model demonstrated improved out-of-sample behavior when trained on data incorporating the post-pandemic phase (Fold 6; training: 2017–2024, testing: January–June 2025; *R*^2^ = 0.831, MAE = 88.9). In-sample reconstruction across the full period also indicated adequate descriptive capacity (*R*^2^ = 0.987).

Probabilistic forecasting with quantile-based prediction intervals demonstrated satisfactory calibration, with empirical coverage around the nominal 95

Feature importance analysis suggested that autoregressive components, the post-COVID regime indicator, and decomposed seasonal trend elements were the main contributors to predictive performance, while climatic variables appeared to act as secondary modifiers in this setting. The STL-LightGBM framework provided a systematic, comprehensible modeling methodology for influenza surveillance in non-stationary environments. The results indicate that hybrid learning based on decomposition may provide a useful complementary alternative to linear models in the presence of structural changes. Future research might explore integrating additional behavioral or mobility parameters, using various machine-learning frameworks, and leveraging larger datasets. This study did not encompass alternative contemporary forecasting methods, including Prophet and Temporal Fusion Transformer (TFT), within its primary benchmarking scope. This analysis concentrated on the assessment of a hybrid framework that was guided by decomposition under structural regime shifts and moderate sample sizes, with a particular emphasis on interpretability for surveillance applications. Although Prophet and TFT have exhibited exceptional performance in a variety of forecasting scenarios, their implementation frequently benefits from the use of larger datasets and extensive tuning procedures. For future research, it may be beneficial to evaluate these architectures using higher-resolution data and extended time horizons in order to enhance comparative assessments.

## Data Availability

The raw data supporting the conclusions of this article will be made available by the authors, without undue reservation.
